# Histological Study on Digestive System of *Triplophysa yarkandensis* in Saline-Alkali and Freshwater Environments: Adaptive Mechanisms

**DOI:** 10.3390/biology14091187

**Published:** 2025-09-03

**Authors:** Zhengwei Wang, Yichao Hao, Yinsheng Chen, Qing Ji, Tao Ai, Shijing Zhang, Jie Wei, Zhaohua Huang, Zhulan Nie

**Affiliations:** 1 College of Life Science and Technology, Tarim University, Alar 843300, China; 10757232145@stumail.taru.edu.cn (Z.W.); 10757242166@stumail.taru.edu.cn (Y.H.); 10757242165@stumail.taru.edu.cn (Y.C.); beioutianhan@163.com (Q.J.); 120070006@taru.edu.cn (J.W.); 2 Xinjiang Production & Construction Corps Key Laboratory of Protection and Utilization of Biological Resources in Tarim Basin, Alar 843300, China; 3 Xinjiang Production and Construction Corps Aquaculture Technology Promotion General Station, Urumqi 830000, China; taoai202506@163.com; 4 Xinjiang Yutian County Fengze Technology Aquaculture Co., Ltd., Yutian County 848400, China; zhangshijing1111@126.com; 5 Alar Changxin Fisheries Company Limited, Alar 843300, China; zhaohuahuang2025@163.com

**Keywords:** *T. yarkandensis*, digestive histology, saline-alkali tolerance, mucus cell plasticity, hepatopancreatic adaptation

## Abstract

*Triplophysa yarkandensis*, a unique saline-alkali tolerant fish in the Tarim River Basin, exhibits unclear adaptive mechanisms of its digestive system to saline-alkali stressors. This study compared the histological characteristics of the digestive system in fish reared in saline-alkali water (salinity 5.89, alkalinity 125.60) and freshwater. Histological characteristics were analyzed using hematoxylin-eosin staining, and parameters were quantified via Image-Pro Plus software, with statistical comparisons performed using independent sample *t*-tests. Key findings included a 2.7-fold increase in oropharyngeal club cell density (48.50 ± 2.68 vs. 17.80 ± 2.04, *p* < 0.01) with denser stratified squamous epithelium in the saline-alkali group; a 74% increase in esophageal goblet cells (104.42 ± 6.67 vs. 59.94 ± 4.68, *p* < 0.01) alongside a 39% reduction in mucosal fold height; 87%, 24%, and 51% increases in villi number across the foregut, midgut, and hindgut, respectively, with an 84% elevation in midgut goblet cells (*p* < 0.01); and mild vacuolization in the hepatopancreas. Results indicate that *T. yarkandensis* adapts via synergistic strategies of enhanced digestive mucus secretion, epithelial structural optimization, and hepatopancreatic metabolic reprogramming. The coordinated villi proliferation and mucus secretion enhance nutrient absorption and osmotic barrier function, providing a theoretical basis for saline-alkali aquaculture.

## Simple Summary

*Triplophysa yarkandensis*, a saline-alkali tolerant fish in the Tarim River Basin, was studied for digestive system adaptations. Comparing saline-alkali and freshwater groups, key findings included increased club cells, goblet cells, and intestinal villi in saline-alkali fish, plus mild hepatopancreatic vacuolization. These show synergistic strategies aiding adaptation, benefiting saline-alkali aquaculture.

## 1. Introduction

Against the backdrop of global ecological changes and water resource development, the study of environmental adaptation mechanisms in fish inhabiting saline-alkali waters has emerged as a frontier in aquatic physiology and evolutionary biology [1,2]. According to statistics from the Food and Agriculture Organization of the United Nations, approximately 20% of global cultivated land and 30% of natural water bodies are facing salinization challenges [3], which pose critical threats to global fisheries, particularly in arid regions where saline-alkali waters limit aquaculture productivity. Inland water systems such as the Tarim River Basin in northwestern China, shaped by unique geological structures and climatic conditions, have formed extreme aquatic environments characterized by high alkalinity (125.60 ± 25.12) and low salinity (5.89 ± 0.32) [4].

*Triplophysa yarkandensis*, a saline-alkali tolerant fish endemic to the Tarim River Basin [5], has long been the focus of research on fish physiology and ecological adaptability in extreme alkalinity. In recent years, with the global promotion of saline-alkali water resource utilization, the digestive adaptation strategies of such specialized fish have garnered increasing attention due to their potential in sustainable aquaculture [6]. In saline-alkali environments, elevated HCO_3_^−^/CO_3_^2−^ and ionic concentrations not only disrupt osmotic balance but also impose unique challenges on the digestive system [7]—as a dual interface for nutrient absorption and osmoregulation, the digestive tract’s ability to undergo histological plasticity in response to ionic stress remains a key scientific question. Unlike the comparative study of gastrointestinal morphology in *T. strauchii* and *T. tenuis* [8], this research focuses on the histological plasticity of *T. yarkandensis* in response to saline-alkali stress, highlighting adaptive mechanisms specific to extreme aquatic environments in the Tarim River Basin.

Existing studies have revealed interspecific variations in saline-alkali responses: *Phoxinus lagowskii* enhances immune defense under chronic stress [9], while *Oreochromis mossambicus* relies on gill ion pumps for osmotic homeostasis [10]. However, *T. yarkandensis*, adapted to the Tarim River’s extreme waters, lacks systematic histological evidence on whether it employs specialized strategies (e.g., mucus secretion enhancement and epithelial barrier optimization) for alkali tolerance. Notably, its hepatopancreatic stress response—characterized by mild vacuolization rather than necrosis—differs fundamentally from euryhaline species, highlighting an urgent need to decode its histological adaptation mechanisms.

This study compares the digestive system histology of *T. yarkandensis* in saline-alkali (salinity 5.89 ± 0.32, alkalinity 125.60 ± 25.12) and freshwater environments, focusing on mucus-secreting cell dynamics, epithelial architecture in the oropharynx/esophagus/intestine, and hepatopancreatic histopathology. We hypothesize that *T. yarkandensis* employs a “mucus-epithelium-hepatopancreas” collaborative model, integrating digestive mucus secretion, epithelial structural optimization, and metabolic reprogramming for saline-alkali adaptation. This research not only advances our understanding of plateau fish adaptation to extreme environments but also provides a theoretical foundation for genetic improvement and aquaculture technology development in saline-alkali waters.

## 2. Materials and Methods

### 2.1. Experimental Materials

*Triplophysa yarkandensis* were obtained from Changxin Fisheries Co., Ltd., Alar, China. Healthy individuals with consistent body length (8.5 ± 0.5 cm) and weight (12.0 ± 1.0 g) were selected and randomly assigned to a saline-alkali water group (*n* = 10; salinity 5.89 ± 0.32, alkalinity 125.60 ± 25.12) and a freshwater control group (*n* = 10; alkalinity 25.12 ± 3.21, salinity 0.325 ± 0.05).

The salinity and alkalinity parameters of the experimental groups were determined based on in-house hydrological survey data collected by our research team from the natural habitat of *T. yarkandensis*. This fish species is mainly distributed in saline-alkali reaches of the Tarim River Basin in Xinjiang, where our field monitoring (conducted from March 2023 to February 2024) shows its native environment has a salinity range of 5.5–6.2 and alkalinity of 110–140. Thus, the saline-alkali group was set to simulate its natural habitat. The freshwater control group parameters refer to our measured water quality data from freshwater tributaries in the basin, where a transition zone between saline-alkali and freshwater exists, and fish encounter such low-salinity and low-alkalinity environments during natural migration. All experimental protocols were approved by the Animal Ethics Committee of Tarim University (approval code, PB20241227001; approval date, 27 December 2024) and complied with the Guidelines for the Ethical Review of Welfare of Laboratory Animals.

Fish were cultured in 100-L recirculating aquaculture systems (RAS, Model RAS-100, Huahai Aquatic Equipment Co., Ltd., Qingdao, China) from 1 May 2024 to 1 November 2024 (6 months), with environmental conditions controlled as follows: water temperature was maintained at 18–22 °C using submersible heaters (Model HT-200, Haier Aquatic Technology Co., Ltd., Qingdao, China), dissolved oxygen >6 mg/L via air pumps (Model AP-300, Boyu Aquatic Supplies Co., Ltd., Guangzhou, China), and pH stabilized using sodium bicarbonate buffer (Food grade, Sinopharm Chemical Reagent Co., Ltd., Shanghai, China), and fish were fed commercial feed (Product Code F-38, Tianbang Aquafeed Co., Ltd., Nantong, China; crude protein 38.5 ± 0.5%, crude fat 8.2 ± 0.3%, fiber 3.1 ± 0.2%) twice daily (09:00 and 18:00) at 3–5% of body weight and fasted for 24 h prior to sampling. Alkalinity was measured by acid–base titration, and salinity was determined using a conductivity meter (Model DDS-307A, INESA Scientific Instrument Co., Ltd., Shanghai, China). Salinity measurements were calibrated with a 1413 μS/cm standard solution prior to use. Alkalinity titration was performed with 0.01 mol/L hydrochloric acid, with the endpoint determined at pH 4.5 using a calibrated pH meter (Model PHS-3C, INESA Scientific Instrument Co., Ltd., Shanghai, China).

### 2.2. Tissue Collection and Processing

At the end of the experiment (09:00–11:00 a.m.), all 10 fish per group were anesthetized with 35 mg/L MS-222 (Fujian Jinjiang Fisheries Co., Ltd., Jinjiang, China) following standard protocols for fish anesthesia [11]. Following loss of equilibrium, the digestive system (oropharyngeal cavity, esophagus, cardia, stomach, pylorus, foregut, midgut, hindgut, liver, and pancreas) was dissected immediately. Tissues were fixed in 4% paraformaldehyde (Shanghai Macklin Biochemical Technology Co., Ltd., Shanghai, China) at room temperature for 48 h; dehydrated through a gradient of 70%, 80%, 90%, 95%, and 100% ethanol (Analytical grade, Sinopharm Chemical Reagent Co., Ltd., Shanghai, China); cleared with xylene (Shanghai Macklin Biochemical Technology Co., Ltd., Shanghai, China); embedded in paraffin (Shanghai Yuanye Bio-Technology Co., Ltd., Shanghai, China); sectioned at 5–7 μm thickness; and stained with hematoxylin and eosin (HE) (Hematoxylin, Shanghai Yuanye Bio-Technology Co., Ltd., Shanghai, China; Eosin, Shanghai Macklin Biochemical Technology Co., Ltd., Shanghai, China) for histological examination [8].

### 2.3. Histological Analysis

Sections were observed using an Olympus BX53 microscope (Olympus Corporation, Tokyo, Japan). For each sample, five random fields of view were selected, and parameters including mucosal fold height and muscular layer thickness in digestive tract segments were measured using Image-Pro Plus software (Version 6.0, Media Cybernetics, Inc., Rockville, MD, USA). Hepatocyte vacuolization in liver tissue was evaluated semi-quantitatively [8].

### 2.4. Statistical Analysis

Data are presented as mean ± standard deviation (SD). All data passed the Shapiro–Wilk test for normal distribution (*p* > 0.05) and Levene’s test for homogeneity of variance (*p* > 0.05), confirming suitability for independent sample *t*-tests using SPSS 27.0 (IBM Corp., Armonk, NY, USA). Excel (Microsoft Office 2021, Microsoft Corp., Redmond, WA, USA) was used for data organization, and SPSS 27.0 was employed for independent sample *t*-tests (two-tailed) to assess group differences. Cohen’s d was calculated to determine effect size when significant differences (*p* < 0.05) were detected.

## 3. Results

### 3.1. Histological Characteristics of Oropharyngeal Cavity

**Figure 1:**
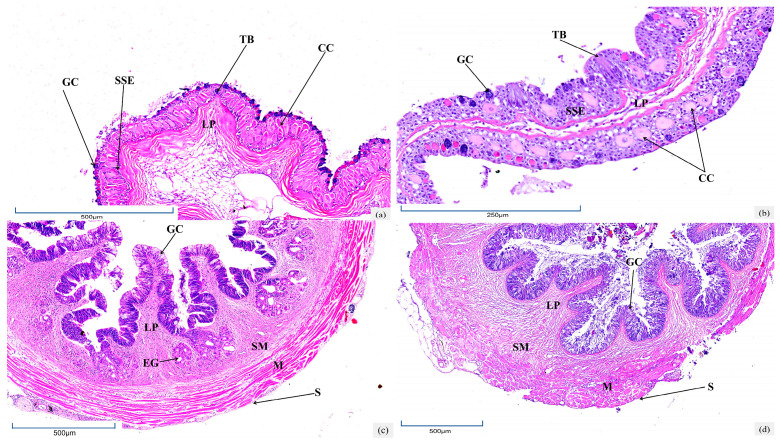

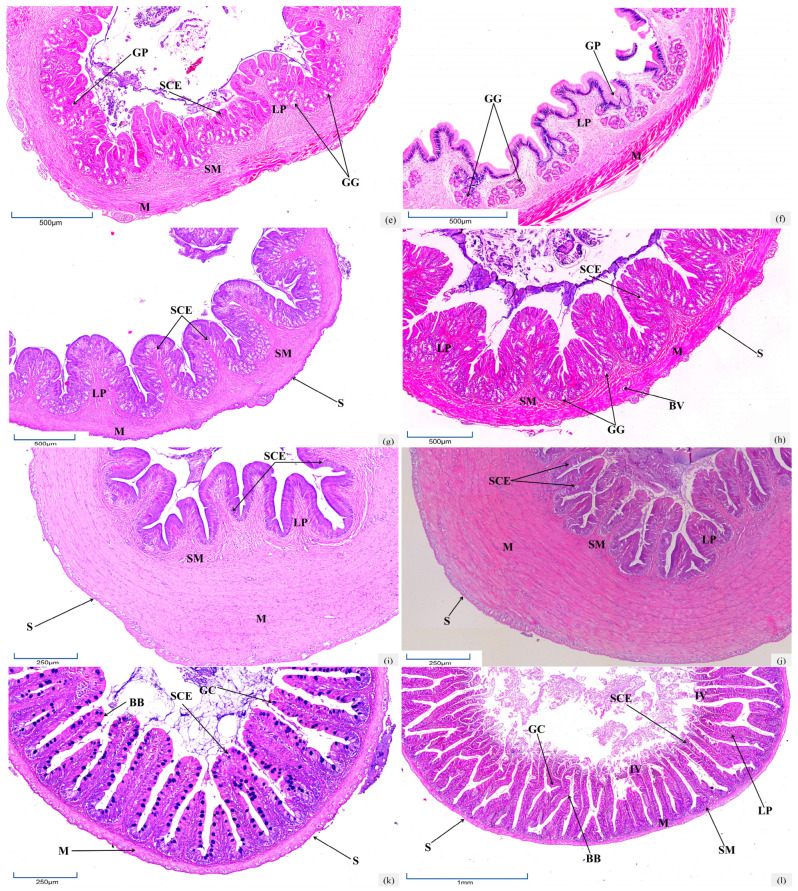

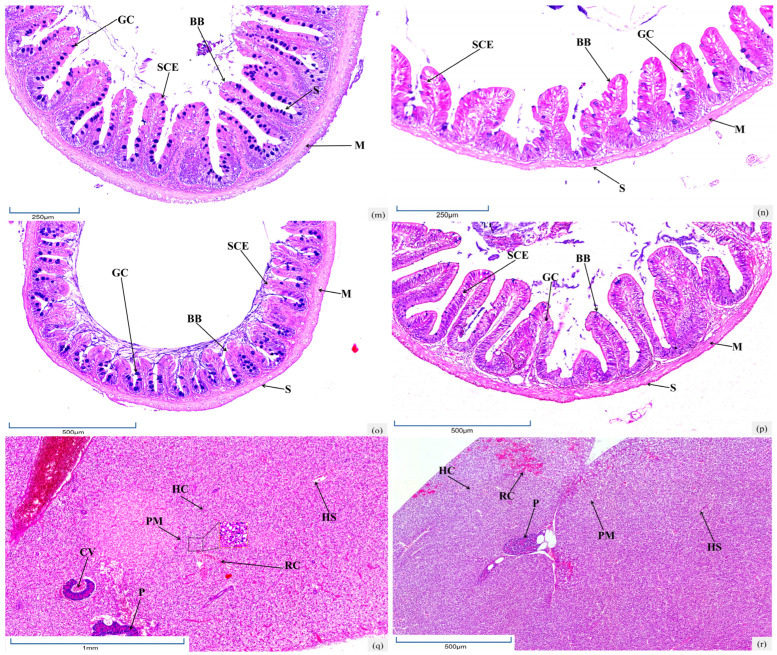
Histological structures of the digestive system in the saline-alkali water group and freshwater group of *T. yarkandensis*: (**a**,**c**,**e**,**g**,**i**,**k**,**m**,**o**,**q**) represent the oropharyngeal cavity, esophagus, cardia, stomach, pylorus, foregut, midgut, hindgut, and hepatopancreas of the saline-alkali water group, respectively; (**b**,**d**,**f**,**h**,**j**,**l**,**n**,**p**,**r**) represent the oropharyngeal cavity, esophagus, cardia, stomach, pylorus, foregut, midgut, hindgut, and hepatopancreas of the freshwater group, respectively. TB—taste bud; M—muscular; LP—lamina propria; GC—goblet cell; SSE—stratified squamous epithelium; CC—club cell; SM—submucosa; EG—esophageal gland; S—serosa; GP—gastric pit; GG—gastric gland; SCE—single columnar epithelium; HS—hepatic sinusoid; PM—hepatic macrophages; RC—red blood cells; P—pancreas; HC—hepatocyte; CV—central vein; BB—brush border; IV—intestinal villus; BV—blood vessels.

The oropharyngeal cavity of *T. yarkandensis*, located at the anterior end of the digestive tract, is composed of mucosa, submucosa, and a muscular layer. The mucosal layer consists of stratified squamous epithelium containing numerous club cells, which are distributed among epidermal cells and significantly larger in volume than other cells. Scattered bottle-shaped taste buds penetrate the epithelium, opening apically into the oropharyngeal cavity and supported by basal lamina elevations. The submucosa is formed by loose connective tissue, while the muscular layer is composed of striated muscles surrounding the wall, interspersed with adipose tissue, blood vessels, and connective tissue. The density of club cells per 100 μm in the saline-alkali group (48.50 ± 2.68) was 2.7-fold higher than that in the freshwater control group (17.80 ± 2.04, *p* < 0.01, Cohen’s d = 3.21; Table 1). Additionally, stratified squamous epithelial cells were more densely arranged in the saline-alkali group, whereas the epithelium was relatively sparse in the freshwater group. Additionally, taste buds in the freshwater group protruded outside the mucosal layer, whereas those in the saline-alkali group were embedded within the mucosal layer (Figure 1a,b).

### 3.2. Histological Characteristics of Esophagus

The digestive tract wall of *T. yarkandensis* from the esophagus consists of four layers: serosa, the muscular layer, submucosa, and mucosa. The serosa is composed of mesothelial cells and thin connective tissue. The muscular layer comprises circumferential striated muscle fibers interspersed with adipose tissue, blood vessels, and connective tissue. The submucosa is formed by loose connective tissue with longitudinal skeletal muscle fibers extending to the lamina propria. In the mucosal layer, goblet cell density in the saline-alkali group (104.40 ± 6.67) increased by 74% compared with the freshwater group (59.90 ± 4.68, *p* < 0.01), but mucosal fold height (reduced by 39%), fold width, muscular layer thickness, and submucosal thickness were all significantly lower (Table 1 and Figure 1c,d).

### 3.3. Histological Characteristics of Cardia, Stomach, and Pylorus

The stomach of *T. yarkandensis* is divided into cardia, fundus, and pylorus. The mucosal epithelium comprises closely arranged simple columnar cells with basal nuclei, and no goblet cells were observed. From cardia to pylorus, the mucosal fold height in both groups first increased then decreased, while fold width in the saline-alkali group showed a reverse trend (decrease then increase). Gastric glands were predominantly long-elliptical, with some extending into slender tubules, and their number was significantly higher in the saline-alkali group. Submucosal thickness decreased from cardia to pylorus in both environments. The muscular layer, composed of inner circular and outer longitudinal smooth muscles, was thickest at the cardia (Table 1 and Figure 1e–j).

### 3.4. Histological Characteristics of Intestine

The intestinal wall of *T. yarkandensis* includes mucosa, submucosa, the muscular layer, and serosa. In the foregut, the mucosa forms dense intestinal villi projecting into the lumen. Villus counts in the foregut, midgut, and hindgut of the saline-alkali group increased by 87%, 24%, and 51%, respectively, versus the freshwater group. The intestinal epithelium in both groups comprised simple columnar absorptive cells with randomly distributed vacuolated goblet cells, whose numbers decreased from foregut to hindgut. As the intestine extended posteriorly, mucosal fold height in the saline-alkali group gradually reduced, with epithelial nuclei remaining basal and a striated border forming at the free end. The columnar cell height and density decreased sequentially. Midgut goblet cell numbers in the saline-alkali group (75.20 ± 4.30) showed an 84% increase compared with the freshwater group (12.76 ± 1.24, *p* < 0.01). The muscular layer thickness continuously decreased in the saline-alkali group, while it first decreased and then increased in the freshwater group (Table 1 and Figure 1k–p).

### 3.5. Histological Characteristics of Hepatopancreas

The hepatopancreas of *T. yarkandensis* is composed of hepatic lobules with circular hepatocytes, whose boundaries are indistinct due to underdeveloped interlobular connective tissue. Central veins within lobules are radially arranged, surrounded by cord-like hepatic plates of polygonal hepatocytes with interspersed hepatic sinuses. Irregular central veins show openings to sinuses, and hepatocyte nuclei are central with distinct nucleoli. Hepatic sinus endothelial cells are flat, with few Kupffer cells and blood cells in the lumen. In the freshwater group, hepatocytes were orderly arranged with clear lobular structures, whereas mild vacuolization occurred in some hepatocytes of the saline-alkali group (Table 1 and Figure 1q,r).

## 4. Discussion

### 4.1. Adaptive Remodeling of Digestive Tract Structure and Enhancement of Barrier Function

The present study revealed significant histological plasticity in the digestive system of *T. yarkandensis* in response to saline-alkali stress, with baseline data (Supplementary Table S1) confirming no initial differences between groups, ensuring that observed changes were induced by environmental factors. In the oropharyngeal cavity, the 2.7-fold increase in club cell density in the saline-alkali group (48.50 ± 2.68 vs. baseline 18.20 ± 2.15 cells/100 μm) highlights a key adaptive strategy, with mucopolysaccharides secreted by these cells forming a protective mucus layer that mitigates epithelial damage from high saline-alkali environments via physical barrier effects [12]. Concurrently, stratified squamous epithelial cells exhibited denser arrangement, analogous to *Cyprinus carpio*’s strategy of enhancing barrier function through epithelial compactness [13], effectively reducing ionic osmotic pressure. Notably, unlike *Mugil cephalus*, which undergoes digestive epithelial atrophy/desquamation under saline-alkali stress [14], *T. yarkandensis* maintains barrier integrity through club cell proliferation and epithelial structural reinforcement, likely rooted in its evolutionary adaptation to northwestern saline-alkali habitats. This epithelial remodeling may also be associated with the upregulation of tight junction proteins (e.g., claudin-1), as reported in *Fundulus heteroclitus* [15]; however, direct evidence for this mechanism in *T. yarkandensis* requires further molecular validation.

The esophageal mucosa displayed a trade-off of “decreased fold height but increased goblet cells”: The esophagus exhibited a trade-off mucosal fold height decreased by 39% (220.13 ± 36.68 vs. baseline 358.70 ± 42.15 μm) to minimize exposed surface area, while goblet cell density increased by 74% (104.42 ± 6.67 vs. baseline 61.30 ± 5.22 cells/100 μm), consistent with enhanced esophageal mucus secretion in *Oreochromis niloticus* under similar stress [16]. Beyond lubrication, mucin in the mucus layer may facilitate osmoregulation by binding divalent cations (e.g., Ca^2+^ and Mg^2+^), as inferred from in vitro studies showing mucin’s ion chelation capacity [17]. This mechanism could reduce ionic influx through the esophageal epithelium, complementing gill-based Na^+^/K^+^-ATPase activity in maintaining osmotic balance. The decrease in fold height might also minimize epithelial surface area exposed to high-alkali water, a trade-off between absorption and protection.

Gastric adaptation was characterized by a 52% increase in gastric gland density (saline-alkali vs. freshwater, *p* < 0.01), aligning with the general mechanism of aquatic organisms augmenting digestive enzyme secretion (e.g., pepsinogen) via gastric gland hyperplasia under saline-alkali stress [18]. The mucosal fold dynamics from cardia to pylorus—with a peak at the fundus in both groups—suggest *T. yarkandensis* prolongs food retention through gastric wall structural optimization, like *Mugil cephalus*’s adaptive gastric motility adjustments to salinity fluctuations [19]. Prolonged retention may enhance nutrient absorption and allow more time for digestive enzymes to neutralize alkaline contents, although direct measurements of gastric emptying rates are required to validate this hypothesis.

Compared with the baseline (6.90 ± 1.23 villi/field), foregut villi in the saline-alkali group increased by 87% (12.70 ± 1.16 villi/field). Midgut goblet cells surged by 84% (72.50 ± 4.30 vs. baseline 40.20 ± 4.35 cells/100 μm). These changes mirror *Micropterus salmoides*’ villi hyperplasia for absorption area expansion [20] and *Danio rerio*’s mucus-mediated barrier enhancement [21]. The villi elongation in the foregut likely boosts nutrient uptake efficiency. In the midgut, the mucus layer secreted by goblet cells may form a pH buffer zone, as proposed for *Gymnocypris przewalskii* in alkaline lakes [22], reducing damage from high HCO_3_^−^ concentrations.

### 4.2. Stress Response and Metabolic Regulation of Hepatopancreas

Saline-alkali exposure induced mild hepatocyte vacuolization, analogous to the response of *Ctenopharyngodon idella* to nitrite stress [23] but distinct from the hepatic necrosis observed in tilapia under high salinity [24]. Baseline data (Supplementary Table S1) confirmed no pre-existing vacuolization in either group at the start of the experiment, verifying that this phenotype is specifically triggered by saline-alkali stress rather than individual variation This mild vacuolization may serve as a marker of cellular stress adaptation: the underdeveloped connective tissue between hepatic lobules in *T. yarkandensis* might render the liver more susceptible to environmental stress yet simultaneously enable faster ion exchange through hepatic sinusoids. In contrast to the euryhaline *Oreochromis mossambicus*, which exhibits acute hepatic inflammation under salinity stress [25], the reversible vacuolization strategy of *T. yarkandensis* likely reflects evolutionary optimization for long-term alkali tolerance.

### 4.3. Molecular Mechanisms Underlying Digestive System Adaptation

The histological plasticity observed in *T. yarkandensis* is likely driven by the coordinated regulation of ion transport genes and stress-responsive signaling pathways, as discussed within the current referenced document. For instance, upregulation of the Na^+^/HCO_3_^−^ co-transporter (NBC) gene in intestinal epithelial cells enhances HCO_3_^−^ excretion to counteract alkaline stress—a mechanism analogous to that reported in *O. niloticus* within the current referenced document [16]. Meanwhile, the increased density of club cells and goblet cells suggests a potential correlation with changes in mucin secretion [26]. However, since this study did not employ mucin-specific histochemical staining (e.g., Periodic Acid-Schiff staining) to directly characterize mucin content, our inference about elevated mucin secretion is based on similar findings. Recent studies on teleost fish included in the current referenced document have shown that the mitogen-activated protein kinase (MAPK) signaling pathway mediates epithelial cell proliferation in response to environmental stress [27], which may explain the villus hyperplasia in the foregut and midgut of *T. yarkandensis*.

Transcriptomic analysis of the hepatopancreas may reveal metabolic reprogram ming toward an energy-conserving mode. The mild vacuolization phenotype likely reflects enhanced autophagy or lipid droplet accumulation for energy storage, like the hepatic adaptation of *C. carpio* under hypoxic conditions [28]. Additionally, upregulation of antioxidant enzymes (e.g., superoxide dismutase [SOD] and catalase [CAT]) in hepatocytes mitigates oxidative damage induced by ionic stress, as documented in *Carassius auratus* exposed to saline-alkali water [29]. These molecular mechanisms likely act in concert to enable *T. yarkandensis* to maintain digestive function without severe tissue damage in extreme environments.

In summary, this study reveals three key adaptive strategies of *T. yarkandensis*: (1) enhancing mucus secretion through increased club cells and goblet cells to strengthen the osmotic barrier, (2) optimizing digestive tract structures (such as villus proliferation) to maintain nutrient absorption efficiency, and (3) reversible hepatopancreatic vacuolization as a metabolic adaptation to saline-alkali stress. These strategies collectively ensure the survival of this species in the extreme environment of the Tarim River and provide a histological basis for saline-alkali aquaculture.

## 5. Conclusions

This study reveals that *T. yarkandensis* adapts to saline-alkali water through digestive tract structural remodeling (2.7-fold increase in oropharyngeal club cells, 74% rise in esophageal goblet cells, and 87% proliferation in foregut villi) and reversible hepatopancreatic vacuolization. These synergistic adaptations enhance mucus secretion, epithelial integrity, and hepatic metabolic regulation, providing critical histological targets for saline-alkali aquaculture.

## Figures and Tables

**Table 1:** Histological parameters of the digestive system of *T. yarkandensis* in saline-alkali and freshwater environments (mean ± SD).

Morphological Index	Group	Oropharyngeal Cavity	Esophagus	Cardia	Stomach	Pylorus	Foregut	Midgut	Hindgut
Club cell (cell number/100 μm)	Saline-alkali water	48.50 ± 2.68 ^a^	-	-	-	-	-	-	-
	Freshwater	17.80 ± 2.04 ^c^	-	-	-	-	-	-	-
Mucosal fold height/μm	Saline-alkali water	-	220.13 ± 36.68 ^a^	196.53 ± 84.95 ^a^	367.56 ± 66.07 ^a^	178.69 ± 21.12 ^a^	677.31 ± 24.60 ^a^	219.07 ± 41.07 ^a^	125.40 ± 7.92 ^a^
	Freshwater	-	360.93 ± 115.51 ^c^	290.91 ± 31.85 ^c^	419.91 ± 64.04 ^c^	185.24 ± 17.48 ^c^	591.17 ± 10.05 ^c^	158.10 ± 15.05 ^c^	313.11 ± 36.59 ^c^
Mucosal fold width/μm	Saline-alkali water	-	63.42 ± 20.86 ^a^	77.79 ± 28.89 ^a^	57.33 ± 5.21 ^a^	71.65 ± 17.56 ^a^	9.06 ± 2.47 ^a^	13.15 ± 3.62 ^a^	6.71 ± 1.44 ^a^
	Freshwater	-	158.04 ± 55.29 ^c^	113.33 ± 12.04 ^c^	68.79 ± 4.90 ^c^	38.62 ± 9.18 ^c^	12.40 ± 2.33 ^c^	10.20 ± 0.90 ^c^	13.10 ± 4.09 ^c^
Submucosa thick/μm	Saline-alkali water	-	29.06 ± 4.97 ^a^	79.52 ± 15.63 ^a^	68.02 ± 10.02 ^a^	20.62 ± 2.79 ^a^	6.73 ± 0.85 ^a^	8.92 ± 1.21 ^a^	9.22 ± 0.58 ^a^
	Freshwater	-	48.09 ± 6.19 ^c^	58.47 ± 10.17 ^c^	52.77 ± 4.06 ^c^	26.47 ± 4.24 ^c^	9.75 ± 2.02 ^c^	5.00 ± 1.36 ^c^	9.59 ± 0.61 ^c^
Muscle layer thickness/μm	Saline-alkali water	-	102.61 ± 11.88 ^a^	156.02 ± 33.23 ^a^	91.28 ± 4.29 ^a^	475.47 ± 41.51 ^a^	27.30 ± 2.71 ^a^	26.40 ± 1.62 ^a^	26.15 ± 3.57 ^a^
	Freshwater	-	125.12 ± 48.96 ^c^	89.26 ± 6.88 ^c^	97.05 ± 3.38 ^c^	399.57 ± 32.85 ^c^	18.67 ± 0.98 ^c^	12.76 ± 1.24 ^c^	17.27 ± 1.31 ^c^
Goblet cell (cell number/100 μm)	Saline-alkali water	-	104.42 ± 6.67 ^a^	-	-	-	82.00 ± 5.58 ^a^	72.50 ± 4.30 ^a^	21.50 ± 3.03 ^a^
	Freshwater	-	59.94 ± 4.68 ^c^	-	-	-	41.20 ± 2.74 ^c^	39.40 ± 4.22 ^c^	19.90 ± 1.85 ^c^
The quantity of intestinal villi	Saline-alkali water	-	-	-	-	-	12.70 ± 1.16 ^a^	12.60 ± 1.35 ^a^	12.40 ± 0.97 ^a^
	Freshwater	-	-	-	-	-	6.80 ± 1.48 ^c^	10.20 ± 1.32 ^c^	8.20 ± 0.63 ^c^

Note: Data are presented as mean ± standard deviation (mean ± SD). For the same morphological index within the same column, values with different superscript letters indicate extremely significant differences between groups (*p* < 0.05, independent samples *t*-test).

## Data Availability

Because the project is not finalized, a link to the data has not been made public.

## Supplementary Materials

The following supporting information can be downloaded at https://www.mdpi.com/article/10.3390/biology14091187/s1: Table S1: Baseline histological parameters of digestive system in *T. yarkandensis* before experiment initiation (*n* = 10 per group).
