# Supplementary material for: Histological Study on Digestive System of Triplophysa yarkandensis in Saline-Alkali and Freshwater Environments: Adaptive Mechanisms
Source: Biology (Basel). 2025 Sep 3;14(9):1187. doi: 10.3390/biology14091187 (PMC12467716; doi:10.3390/biology14091187)
Supplement: Supplementary file 1 [file biology-14-01187-s001.zip › biology-3797900-supplementary.pdf]

biology

Supplementary Materials

Table S1. Baseline histological parameters of digestive system in *T. yarkandensis* before experiment initiation ($n = 10$ per group).

Histological Parameter	Saline-Alkali Group (Initial)	Freshwater Control Group (Initial)	Intergroup Difference
Oropharyngeal club cell density (cells/100μm)	18.20 ± 2.15	17.90 ± 1.98	$p > 0.05$
Esophageal goblet cell density (cells/100μm)	61.30 ± 5.22	59.70 ± 4.85	$p > 0.05$
Foregut villus count (number/field)	6.90 ± 1.23	6.70 ± 1.15	$p > 0.05$
Midgut villus count (number/field)	10.30 ± 1.32	10.10 ± 1.28	$p > 0.05$
Hindgut villus count (number/field)	8.30 ± 0.76	8.10 ± 0.69	$p > 0.05$
Midgut goblet cell density (cells/100μm)	40.20 ± 4.35	39.50 ± 3.92	$p > 0.05$

Note: Data are presented as mean ± standard deviation (mean ± SD). No significant differences were detected between groups at the initiation of the experiment (independent samples t-test, $p > 0.05$).

 https://doi.org/10.3390/biology14091187
